# Pancreatic stellate cell‐derived exosomal tRF‐19‐PNR8YPJZ promotes proliferation and mobility of pancreatic cancer through AXIN2

**DOI:** 10.1111/jcmm.17852

**Published:** 2023-07-24

**Authors:** Wenpeng Cao, Shisi Dai, Wanyuan Ruan, Tingting Long, Zhirui Zeng, Shan Lei

**Affiliations:** ^1^ Department of Anatomy, School of Basic Medicine Guizhou Medical University Guiyang China; ^2^ Department of Anatomy, School of Basic Medicine Guizhou Nursing Vocational college Guiyang China; ^3^ School of Clinical Medicine Guizhou Medical University Guiyang China; ^4^ Department of Physiology, School of Basic Medicine Guizhou Medical University Guiyang China

**Keywords:** AXIN2, exosomal, metastasis, pancreatic cancer, tRF‐19‐PNR8YPJZ

## Abstract

The pancreatic stellate cells (PSCs) play an important role in the development of pancreatic cancer (PC) through mechanisms that remain unclear. Exosomes secreted from PSCs act as mediators for communication in PC. This study aimed to explore the role of PSC‐derived exosomal small RNAs derived from tRNAs (tDRs) in PC cells. Exosomes from PSCs were extracted and used to detect their effects on PC cell proliferation, migration and invasion. Exosomal tDRs profiling was performed to identify PSC‐derived exosomal tDRs. ISH and qRT‐PCR were used to examine the tRF‐19‐PNR8YPJZ levels and clinical value in clinical samples. The biological function of exosomal tRF‐19‐PNR8YPJZ was determined using the CCK‐8, clone formation, wound healing and transwell assays, subcutaneous tumour formation and lung metastatic models. The relationship between the selected exosomal tRF‐19‐PNR8YPJZ and AXIN2 was determined by RNA sequencing, luciferase reporter assay. PSC‐derived exosomes promoted the proliferation, migration, and invasion of PC cells. Novel and abundant tDRs are found to be differentially expressed in PANC‐1 cells after treatment with PSC‐derived exosomes, such as tRF‐19‐PNR8YPJZ. PC tissue samples showed markedly higher levels of tRF‐19‐PNR8YPJZ than normal controls. Patients with PC exhibiting high tRF‐19‐PNR8YPJZ expression had a highly lymph node invasion, metastasis, perineural invasion, advanced clinical stage and poor overall survival. Exosomal tRF‐19‐PNR8YPJZ from PSCs targeted AXIN2 in PC cells and decreased its expression, thus activating the Wnt pathway and promoting proliferation and metastasis. Exosomal tRF‐19‐PNR8YPJZ from PSCs promoted proliferation and metastasis in PC cells via AXIN2.

## 1 INTRODUCTION

Pancreatic cancer (PC) is considered the ‘king’ among malignancies as its 5‐year survival rate is less than 5%.^1^, ^2^ Additionally, early metastasis and rapid invasion commonly occur in patients with PC.^3^ Although curative resection is the most common treatment for PC, only 15%–20% of patients benefit from it in the early stages.^4^ Furthermore, most patients have PC recurrence within 2 years after resection, so understanding the molecular mechanisms involved in PC development may aid in diagnosis and treatment.

Stellate cells of the pancreas (PSCs) are stromal cells that contribute to the cancer microenvironment in PC tissue.^5^ PSCs have been found to be involved in PC cell invasion and metastasis in several studies.^6^, ^7^ Exosomes, small membranous vesicles, are mediators secreted by PSCs that affect PC progression. Studies have shown that PSC‐derived exosomes promote PC proliferation, migration, and drug resistance by delivering proteins, mRNA, and non‐coding RNA, leading to changes in gene profiles and activation of oncogenic pathways.^8^, ^9^, ^10^ It has been reported that small ncRNAs can be derived from the precursors and mature sequences of small RNAs derived from tRNAs (tDRs).^11^, ^12^ RNA polymerase III transcribes tRNAs, which are typically 76–90 nucleotides long in eukaryotes. It has been found that pre‐tRNAs and mature tRNAs are extensively modified before and after they are exported to the cytoplasm in order to create two different kinds of can generally be divided into tRNA halves (tiRNAs) and tRNA‐derived fragments (tRFs).^13^, ^14^ Exosomal tDRs have been used as biomarkers in liquid biopsies to differentiate cancer patients from healthy controls.^15^ However, it is still unclear how tDRs regulate the biological properties of malignant PC.

In the present study, we detected the role of PSC‐derived exosomal tDRs in PC. We demonstrated that PSCs secreted exosomal tRF‐19‐PNR8YPJZ and delivered them to PC cells, thereby enhancing proliferation and mobility via regulating AXIN2. Our present study suggests that exosomal tRF‐19‐PNR8YPJZ may be a potential biomarker and an effective target for the diagnosis and clinical therapy of PC.

## 2 MATERIALS AND METHODS

### 2.1 Clinical specimens and ethical statement

The present study used 80 PC and corresponding adjacent non‐tumour tissues. All samples were collected prior to chemotherapy and radiotherapy in patients with PC. All samples were stored at −80°C before performing research. This study was approved by the ethical committee at Guizhou Medical University, which followed the Declaration of Helsinki. Informed consent was obtained from all participants.

### 2.2 Cell culture and lentivirus infection

PSCs (Cat no. CP‐H024), AsPc‐1 (Cat no. CL‐0027) and PANC‐1 (Cat no. CL‐0184) cells were obtained from Procell (https://www.procell.com.cn/; Wuhan, China). PSCs cells were cultured in DMEM/F12 medium (HyClone, USA) containing 10% foetal bovine serum. PC cells including AsPc‐1 and PANC‐1 were cultured in high‐glucose DMEM (HyClone, USA) with 10% FBS (Gibco, USA). All cells were cultured in 5% CO_2_ and 100% humidity at 37°C. tRF‐19‐PNR8YPJZ‐overexpression and tRF‐19‐PNR8YPJZ‐knockdown lentiviruses were obtained from Dongxuan Biotech (Jiangsu, China). Subcloning the PCR‐amplified human AXIN2 and tRF‐19‐PNR8YPJZ cDNA into the pMSCV retrovirus plasmid led to the construction of an overexpression lentivirus. Small interfering RNAs (siRNAs) against AXIN2 were obtained from Shanghai Shenggong Co., Ltd. As instructed by the manufacturer, transient transfections were performed using Lipo2000 (Invitrogen). After lentivirus infection 72 h with polybrene (Thermo Fisher Scientific, USA), stable PC cells were selected for 2 weeks with 0.5 g/mL puromycin (Biomedicine Biotech).

### 2.3 CCK‐8 assay

A density of 4 × 10^3^ AsPc‐1 and PANC‐1 cells per well, with eight parallel wells per group, was applied in 96‐well plates. For exosome treatment group, exosome with concentration as 1 μg/mL was added. Various times (6, 24, 48, 72 and 96 h) of culture in the incubator followed by a 1‐h incubation in fresh medium containing 10% CCK‐8 solution (Dojindo, Japan) was conducted. An automatic multifunctional enzyme labeller (Varioskan LUX, Thermo Fisher Scientific, USA) was used to measure absorption at 450 nm.

### 2.4 Colony formation assay

Three parallel wells were plated with PANC‐1 and AsPC‐1 cells (400 cells/well). For exosome treatment group, exosome with concentration as 1 μg/mL was added. Cells were incubated at 37°C for 14 days, followed by 10 min of methanol fixation and 30 min of crystal violet staining (Solarbio, China). Finally, a camera was used to record the condition of cell colonies in per plate.

### 2.5 Wound healing assay

Cells were grown in three parallel wells of a 6‐well plate. After the cells reached 80%–90% confluency, sterile micropipettes were used to scrape off the cells to create a wound. Fresh medium was added after washing the cells with PBS to remove cell debris. For exosome treatment group, exosome with concentration as 1 μg/mL was added. Three fields of view were selected for each group and photographed to calculate the wound edge distance at 0 and 24 h after scraping.

### 2.6 Transwell assay

Total 300 μL cell suspension with the density of 1 × 10^5^ cells/mL was transferred to the upper chamber (Costar, USA) which pre‐coated with 8% matrigel (Thermo Fisher Scientific, USA). Total 700 μL DMEM containing 10% FBS was set in the lower chambers. For exosome treatment group, exosome with concentration as 1 μg/mL was added in lower chambers. After 48 h, upper chambers were taken out and incubated with 4% paraformaldehyde for 15 min. Then, cotton swabs were used to remove the non‐invasive cells, and then crystal violet was used to stain cells. Under a microscope (magnification times, 100×), three randomly selected views of transwell were recorded and invasive cells were counted.

### 2.7 Isolation of exosomes

The Invitrogen Total Exosome Isolation Kit (ThermoFisher, USA) was used to isolate exosomes from PSCs culture medium. PSCs derived exosomes was isolated by mixing 200 μL culture medium with the precipitation reagent as per manufacturer's instructions. Exosomal RNA was isolated using an exoRNeasy Maxi kit (YEASEN, China).

### 2.8 Transmission electron microscope (TEM)

Exosomes isolated from culture medium were examined using TEM. Copper electron microscopy grids (Servicebio, China) coated with Formvar carbon were dipped into 1% glutaraldehyde once exosomes had been fixed in 1% glutaraldehyde. To stain the exosomes, 2% uranyl oxalate and PBS were added in sequence, followed by air‐drying overnight. Finally, the images of exosomes were photographed using a TEM (Electronics Co. LTD, Japan).

### 2.9 Small RNA‐seq analysis

The manufacturer's instructions were followed for preparing a sequencing library to study tDRs expression, with NEXTflex Small RNA‐seq Kit v3. Raw sequencing data were cleaned by removing 5′ and 3′ adapters, filtering low‐quality reads, merging identical reads and counting the unique reads. Only reads with 16–40 nt insertion were retained for further analysis. To identify tDRs, reads were first aligned to mature tRNAs and downstream sequences using BLAST. Only the reads perfectly matched to the genome were counted and classified into 5′‐, 3′‐, I′‐ and 3′‐U of tRNA according to the positions from which tDRs were generated. To allow quantitative comparisons, the expression level of specific tDR was normalized to the total tDRs. Differentially expressed tDRs were identified using the R package Deseq2. Statistical significance was determined by log‐fold change ≥1 and *p* < 0.05. The differentially expressed tDRs were presented using a heatmap.

### 2.10 Dual‐luciferase reporter gene assay

Wild‐type and site‐mutation AXIN2 luciferase reporter plasmids were constructed by GeneChem (Shanghai, China). Then, these plasmids were transfected into NC and tRF‐19‐PNR8YPJZ overexpression cells. Dual‐Luciferase Reporter Gene Assay Kit from Promega (Madison, WI, USA) was used to measure luciferase activity based on the manufacturer's instructions.

### 2.11 Quantitative real‐time PCR

The total RNA was extracted with TRIzol (Invitrogen, Carlsbad, CA, USA). The cDNA was synthesized using the RiboSCRIPTTM Reverse Transcription Kit (Ruibo, China). Guangzhou Ruibo Biotechnology Co., Ltd. provided the primers used in current study. For qRT‐PCR, SYBR green reagent (Ruibo, China) in combination with a microplate reader (BioTek, Epoch, USA) were used based on the manufacturer's instructions. 2^−∆∆Ct^ method was used to calculate the relative gene expression. Table S1 includes the primer sequences used for qRT‐PCR.

### 2.12 Western blotting

Bicinchoninic acid method was used to measure the protein concentration of each sample. An amount of protein lysate (20 μg) was separated on 10% SDS‐PAGE gels and transferred to PVDF membranes (Merck, USA). The membranes were then incubated with skimmed milk at 25°C for 1 h and then incubated with CD9 (20597‐1‐AP; Proteintech, China), TSG101(28283‐1‐AP; Proteintech, China), ALIX (12422‐1‐AP; Proteintech, China), β‐catenin (51067‐2‐AP; Proteintech, China), AXIN2 (20540‐1‐AP; Proteintech, China), TCF4 (22337‐1‐AP; Proteintech, China), LEF1 (14972‐1‐AP; Proteintech, China) and β‐actin (20536‐1‐AP; Proteintech, China) overnight at 4°C. The membranes were washed by TBST and incubated with secondary antibodies at 25°C for 2 h. An enhanced chemiluminescence reagent (Boster, Wuhan, China) was used to detect the protein bands. Relative expression of proteins in cells were calculated using the internal control as β‐actin. The experiments were performed at least in triplicate and repeated at least three times, and the data are representative of replicate experiments.

### 2.13 Animal experiments

A subcutaneous tumorigenesis model and lung metastasis model were used to study the proliferation and metastasis ability of PC cell in vivo. Before performing injection of PANC‐1 cells into mice, NC and PANC‐1 cells with tRF‐19‐PNR8YPJZ knockdown were pre‐treated with 1 μg/mL PSCs‐derived exosome for 7 days. For the subcutaneous tumorigenesis model, the right flank of the mice was subcutaneously injected with 1 × 10^6^ cells. The health conditions of mice were monitored daily. Their tumour sizes were monitored weekly. Mice were sacrificed on Day 28. Tumour tissues were extracted, weighed and subjected to immunohistochemical staining. Injection of PANC‐1 cells into the caudal vein of mice was used to create the lung metastasis model. The health conditions of mice were monitored daily. Mice were sacrificed when they developed dyspnoea. The lung tissues of mice were extracted and used to calculate metastatic foci. The Guizhou Medical University Animal Ethics Committee approved the experiments with animals (approval number: 2200187).

### 2.14 Statistical analysis

An analysis of all results was performed using GraphPad Prism 9 (GraphPad Software, USA). To identify significant differences between two groups and multigroups, unpaired *t*‐test and one‐way analysis of variance were conducted. Results were presented as mean ± standard error of mean (SEM). The difference was considered significant if *p* < 0.05.

## 3 RESULTS

### 3.1 PSC‐derived exosomes promote cells proliferation and mobility

**FIGURE 1:**
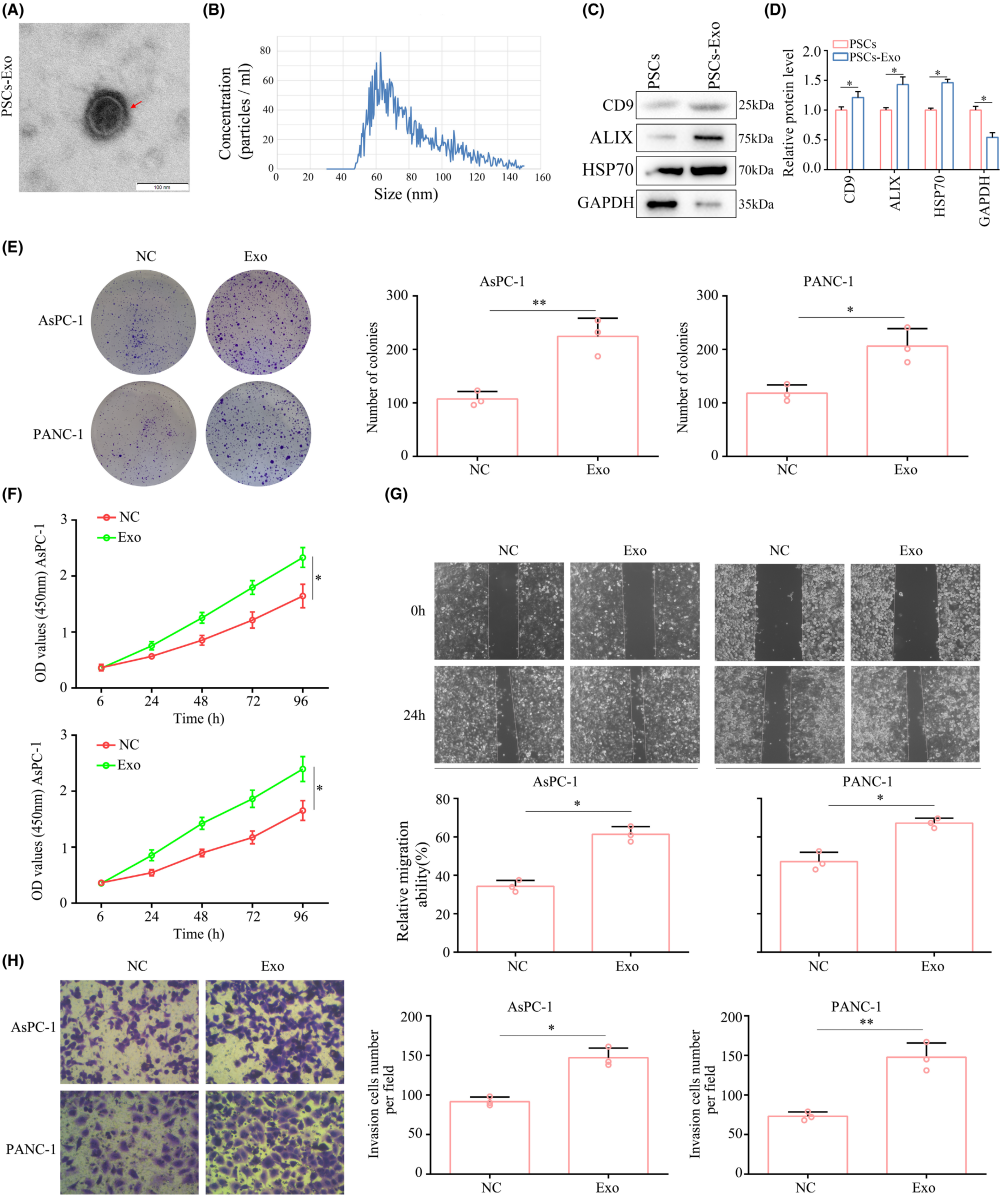
PSC‐derived exosomes promoted the proliferation and mobility of PC cells. (A, B) Electron microscopic characteristics of PSC‐derived exosomes. (C, D) Expression of CD9, ALIX, HSP70 and GAPDH in PSCs and PSC‐derived exosomes. PANC‐1 and AsPC‐1 were treated with PSC‐derived exosomes, while treatment with equivalent medium was set as control. (E) Colony formation assays were used to detect the cell colony growth of PANC‐1 and AsPC‐1 cells. (F) CCK8 assays were used to detect the proliferation of PANC‐1 and AsPC‐1 cells. (G) Wound healing assays were used to detect the migration of PANC‐1 and AsPC‐1 cells. (H) Transwell assays were used to detect the invasion of PANC‐1 and AsPC‐1 cells. *p < 0.05; **p < 0.01.

As the result of TEM detection, PSC‐derived exosomes exhibited the vesicle‐like structure of the bilayer (Figure 1A). The size of PSC‐derived exosomes was most in 50–90 nm (Figure 1B). Actually, PSC‐derived exosomes demonstrated high CD9, ALIX and HSP70 expression (Figure 1C,D). Using PSC‐derived exosomes to treat PC cells, a significant increase in colony formation was observed with PSC‐derived exosomes (Figure 1E) compared with that in the negative control in AsPC‐1 and PANC‐1 cells. The proliferation of AsPC‐1 and PANC‐1 cells was tested using the CCK‐8 assay, AsPC‐1 and PANC‐1 cells treatment with PSC‐derived exosomes exhibited higher proliferation rate (Figure 1F). In addition, both wound‐healing and transwell assays demonstrated that PSC‐derived exosomes significantly enhanced migration and invasion of PC cells (Figure 1G,H).

### 3.2 Identification of prominent exosomal tsRNAs secreted from PSCs

**FIGURE 2:**
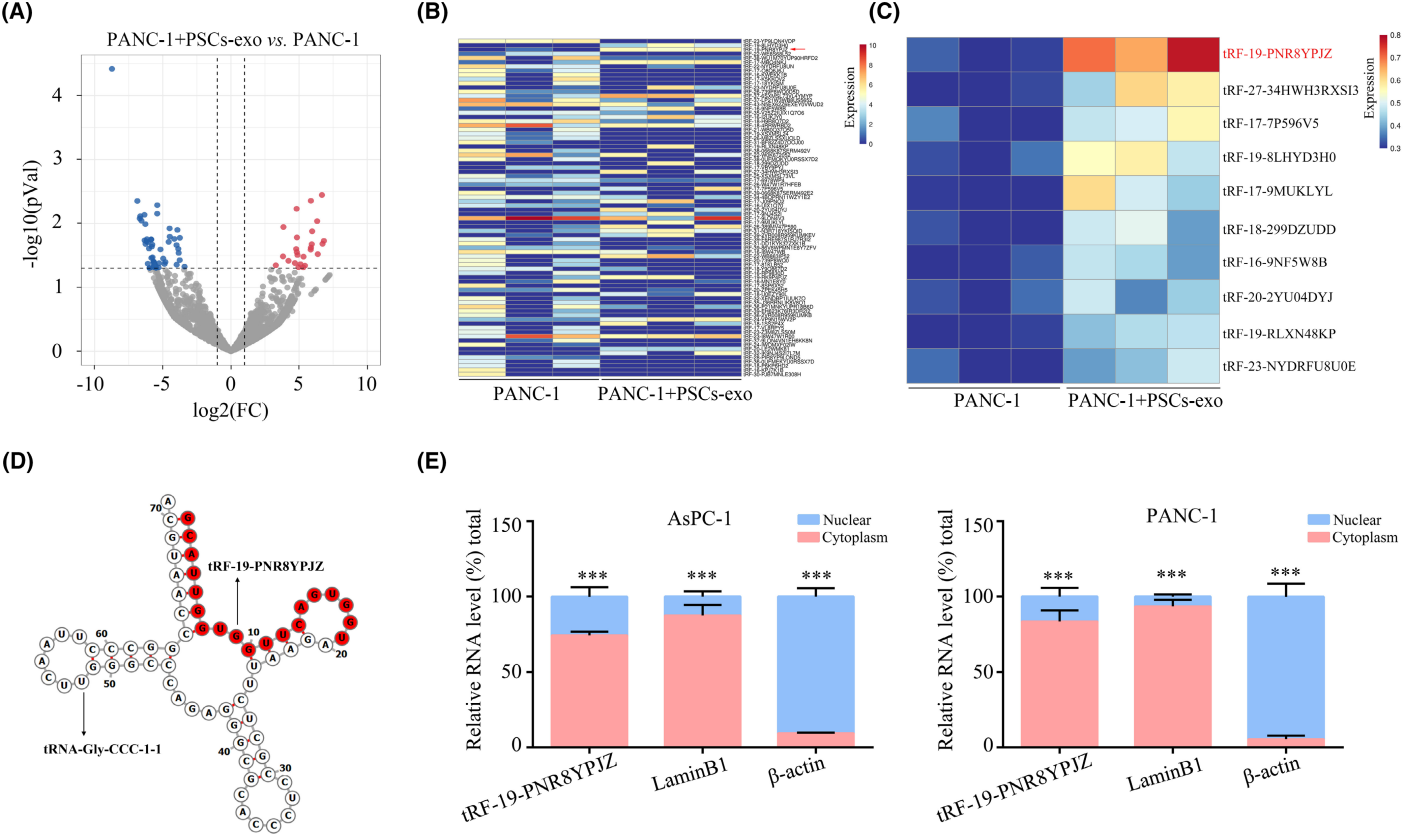
tRF‐19‐PNR8YPJZ was increased in PANC‐1 cells treatment with PSC‐derived exosomes. (A) Volcano Plot showed different expression of exosomal tDRs between PANC‐1 cells with and without PSC‐derived exosomes. (B) Heatmap showed different expression of exosomal tDRs between PANC‐1 cells with and without PSC‐derived exosomes. (C) qRT‐PCR was used to detect the expression between exosomal top 10 tDRs between the PANC‐1 cells with and without PSC‐derived exosomes. (D) tRF‐19‐PNR8YPJZ structure. (E) Nucleocytoplasmic separation assay revealed that tRF‐19‐Q1Q89PJZ is mainly expressed in the cytoplasm. ***p < 0.01.

To identification of the tDRs from PSC‐derived exosomes involved in the progression of PC cells, small RNA‐seq analysis was performed in PANC‐1 cells with/without PSC‐derived exosome treatment. RNA sequencing study revealed that 80 exosomal small RNAs derived from tRNAs (tDRs) were differentially expressed were found (Figure 2A,B). The top 10 significantly changed exosomal tDRs in PANC‐1 were further validated by qRT‐PCR. The results showed that 5′‐tRF‐19‐PNR8YPJZ was upregulated the most in PANC‐1 cells treatment with PSC‐derived exosomes (Figure 2C). OncotRF Database showed tRF‐19‐Q1Q89PJZ has originated from the 5′‐end of mature tRNA‐Gly‐CCC‐2‐2 carrying the anticodon CCC (Figure 2D). MINTbase placed tRF‐19‐PNR8YPJZ in the 19 nt small RNA class with a sequence of 5′‐ GCATTGGTGGTTCAGTGGT‐3′ (Figure S1). Furthermore, nuclear/cytoplasmic RNA quantification exhibited that tRF‐19‐PNR8YPJZ transcription was prominent to the cytoplasm (Figure 2E).

### 3.3 The tRF‐19‐PNR8YPJZ is upregulated in PC

**FIGURE 3:**
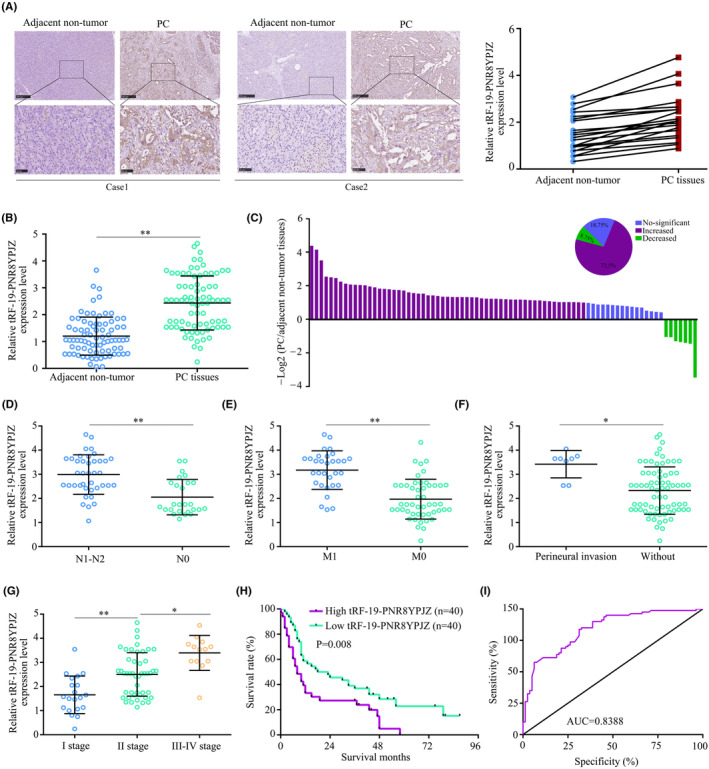
Expression and diagnostic value of tRF‐19‐PNR8YPJZ in clinical sample. (A) ISH analysis of tRF‐19‐PNR8YPJZ expression in PC and adjacent non‐tumour tissues. (B) The expression level of tRF‐19‐PNR8YPJZ was detected by qRT‐PCR in 80 paired PC and adjacent non‐cancerous tissues. (C) The fold changes (log2) of tRF‐19‐PNR8YPJZ in each paired sample were arranged from high to low. (D) Correlation between tRF‐19‐PNR8YPJZ expression and lymph node invasion. (E) Correlation between tRF‐19‐PNR8YPJZ expression and distant metastasis. (F) Correlation between tRF‐19‐PNR8YPJZ expression and perineural invasion. (G) Correlation between tRF‐19‐PNR8YPJZ expression and clinical stage. (H) PC cases were divided into two groups according to the median value of tRF‐19‐PNR8YPJZ expression. Overall survival was analysed by Kaplan–Meier survival analysis using the log‐rank test. (I) Receiver operating curve curve showing the diagnostic sensitivity and specificity of tRF‐19‐PNR8YPJZ in PC. *p < 0.05; **p < 0.01.

Based on ISH and RT‐qPCR analysis in 80 human PC tissues and adjacent non‐cancerous tissues, the expression of tRF‐19‐PNR8YPJZ in tumour tissues was higher than in normal tissues (Figure 3A,B). In 58 (72.5%) of the paired clinical samples, tRF‐19‐PNR8YPJZ was more abundant in tumour tissues than in non‐cancerous tissues (Figure 3C). tRF‐19‐PNR8YPJZ expression in PC patients was correlated with clinicopathological parameters through correlation analysis. Results showed that tRF‐19‐PNR8YPJZ expression was higher in PC tissues with regional lymph node invasion (Figure 3D), metastasis (Figure 3E), perineural invasion (Figure 3F) and an advanced clinical stage (Figure 3G). Age, gender and tumour size did not show any significant differences (Table 1). Additionally, we used the median values of expression levels of tRF‐19‐PNR8YPJZ as cut‐off values for classifying PC tissues with high or low expression levels. tRF‐19‐PNR8YPJZ expression was associated with poor outcome for PC patients in Kaplan–Meier survival analysis (Figure 3H). According to receiver operating curve analysis, the area under the curve was 0.8388, which indicates that tRF‐19‐PNR8YPJZ may be able to diagnose PC (Figure 3I).

**TABLE 1:** Correlation between pancreatic cancer characteristics and tRF‐19‐PNR8YPJZ expression levels.

tRF‐19‐PNR8YPJZ expression					
Features	*n*	Low	High	*χ* ^2^	*p* value
All cases	80	40	40		
Age				1.289	0.256
<60	32	17	15		
≥60	48	23	25		
Gender				1.289	0.256
Man	47	21	26		
Female	33	19	14		
Tumour size (cm)				0.818	0.366
<2	34	15	19		
≥2	46	25	21		
Lymph node metastasis				14.459	<0.001
Negative	41	29	12		
Positive	39	11	28		
TNM stage				7.44	0.006
I and II	67	38	29		
III and IV	13	2	11		
Distant metastasis				5	0.025
Negative	72	39	33		
Positive	8	1	7		
Perineural invasion				32.916	<0.001
Negative	49	37	12		
Positive	31	3	28		

### 3.4 PSC‐derived exosomal tRF‐19‐PNR8YPJZ exhibits stimulative effects on proliferation, migration and invasion of PC cells

**FIGURE 4:**
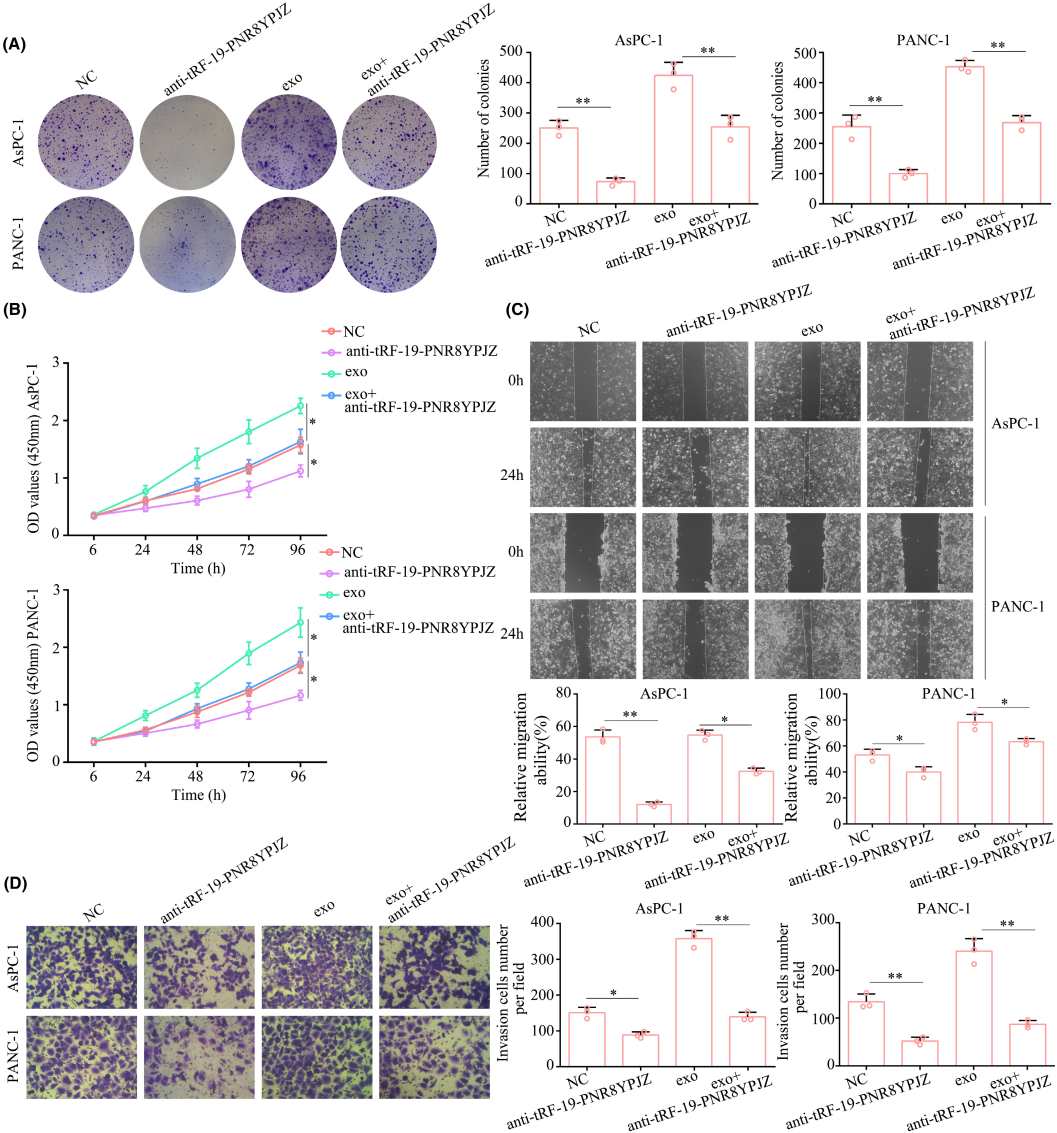
PSC‐derived exosomal tRF‐19‐PNR8YPJZ promoted of PC cells proliferation and mobility. Pancreatic cancer cell PANC‐1 and AsPC‐1 were transfected with tRF‐19‐PNR8YPJZ knockdown lentiviruses (anti‐tRF‐19‐PNR8YPJZ) and negative control (anti‐NC) prior to treatment with PSC‐derived exosomes. (A) Colony formation assays were used to detect the cell colony growth. (B) CCK‐8 was used to detect cell proliferation. (C) Wound healing assay was performed to detect the cell migration. (D) Transwell assays were used to detect the cell invasion. *p < 0.05; **p < 0.01.

Next, we determined whether PSC‐derived exosomal tRF‐19‐PNR8YPJZ promoted proliferation and mobility of PC cells. Before treating PC cells with PSC‐derived exosomes, we transfected them with tRF‐19‐PNR8YPJZ knockdown lentiviral. We investigated the role of tRF‐19‐PNR8YPJZ in cell lines as it is overexpressed and knocked down in PC tissues and cell lines. Stable tRF‐19‐PNR8YPJZ knockdown (anti‐tRF‐19‐PNR8YPJZ) and overexpression (tRF‐19‐PNR8YPJZ) AsPC‐1 and PANC‐1 stable cell lines were constructed by lentiviral transfection (Figure S2). CCK‐8 and colony formation experiments revealed that tRF‐19‐PNR8YPJZ‐inhibition reduced the effects of PSC‐derived PC exosomes on PC proliferation and colony formation (Figure 4A,B). Furthermore, in wound healing and transwell assays, tRF‐19‐PNR8YPJZ knockdown inhibits migration and invasion of PC cells. A similar effect was achieved when PC cells were inhibited tRF‐19‐PNR8YPJZ to reverse the PSC‐derived exosome enhancement of migration and invasion (Figure 4C,D).

### 3.5 PSC‐derived exosomal tRF‐19‐PNR8YPJZ exhibits stimulative effect on PC proliferation and metastasis in vivo

**FIGURE 5:**
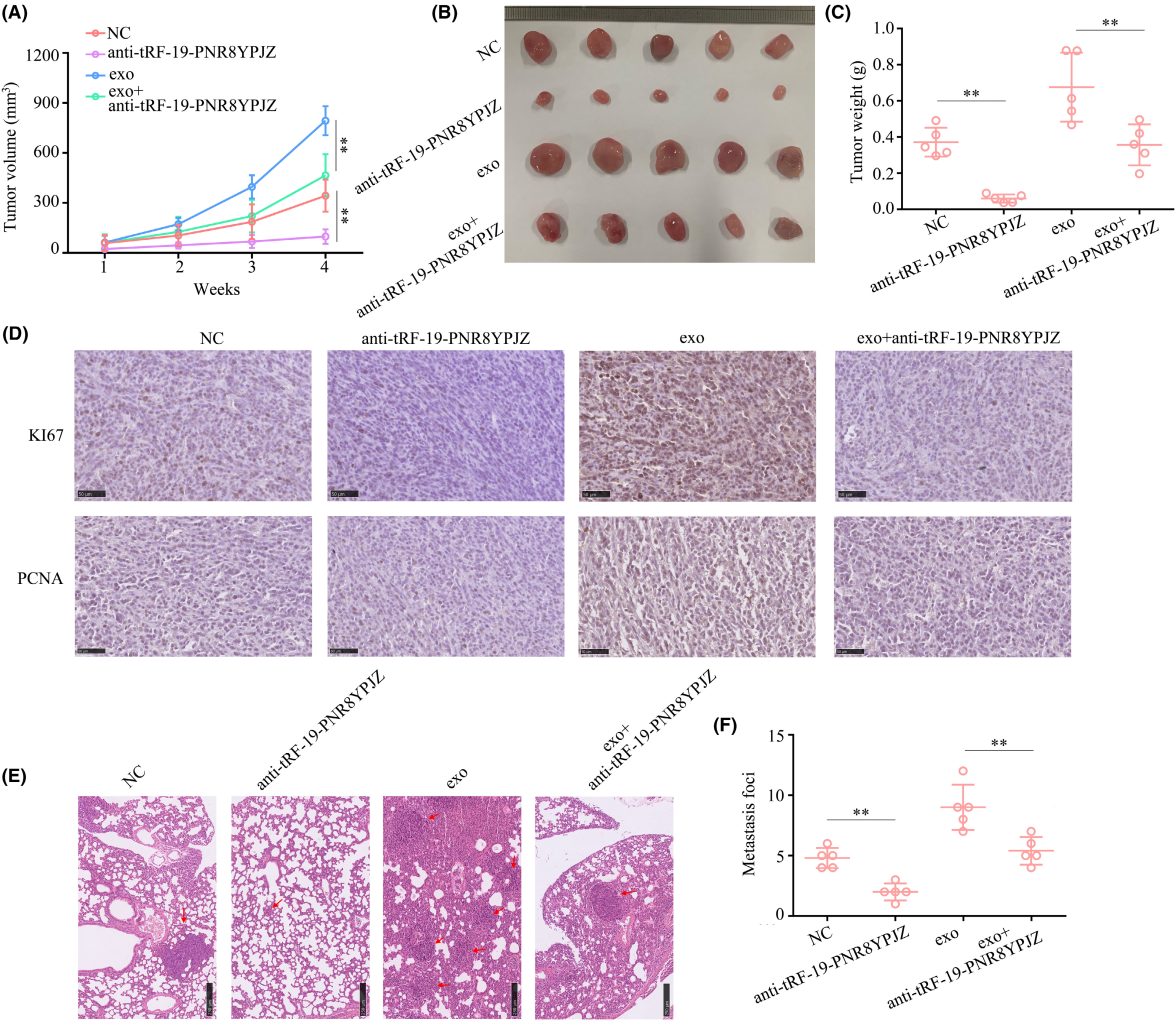
tRF‐19‐PNR8YPJZ promoted the proliferation and metastasis of PC cells in vivo. (A) Subcutaneous tumour volume. (B) A representative image of nude mouse tumours (*n* = 5). (C) Subcutaneous tumour weight. (D) Representative IHC staining images showing Ki‐67 and PCNA expression in transplanted tumours under different experimental conditions. (E, F) Representative IHC staining of HE images showing metastatic loci in lung. **p < 0.01.

To demonstrate the effect of exosomal tRF‐19‐PNR8YPJZ on PC phenotypes in vivo, we pre‐treated tRF‐19‐PNR8YPJZ‐knockdown and control cells with or without PSC‐derived exosomes, and injected them into mice. Subcutaneous xenograft models and lung metastasis models were constructed. Results demonstrated that tumour tissues originating from PANC‐1 cells treated with exosomes grew quickly and were heavier, tRF‐19‐PNR8YPJZ‐knockdown decreased the proliferation of tumour tissues originating from PANC‐1 cells and their weight (Figure 5A–C). Furthermore, tumour tissues originating from PANC‐1 cells treated with exosomes had higher KI67 and PCNA expression than those originating from control cells, whereas KI67 and PCNA expression was reduced in tumour tissues derived from PANC‐1 cells with tRF‐19‐PNR8YPJZ‐knockdown (Figure 5D). The results of the pulmonary metastatic model indicated that PANC‐1 cells treated with exosomes exhibited higher metastatic ability compared with that of control cells in vivo, and lower metastatic foci were observed in the lung tissues of mice injected with tRF‐19‐PNR8YPJZ‐knockdown PANC‐1 cells (Figure 5E,F).

### 3.6 PSC‐derived exosomal tRF‐19‐PNR8YPJZ targets AXIN2

**FIGURE 6:**
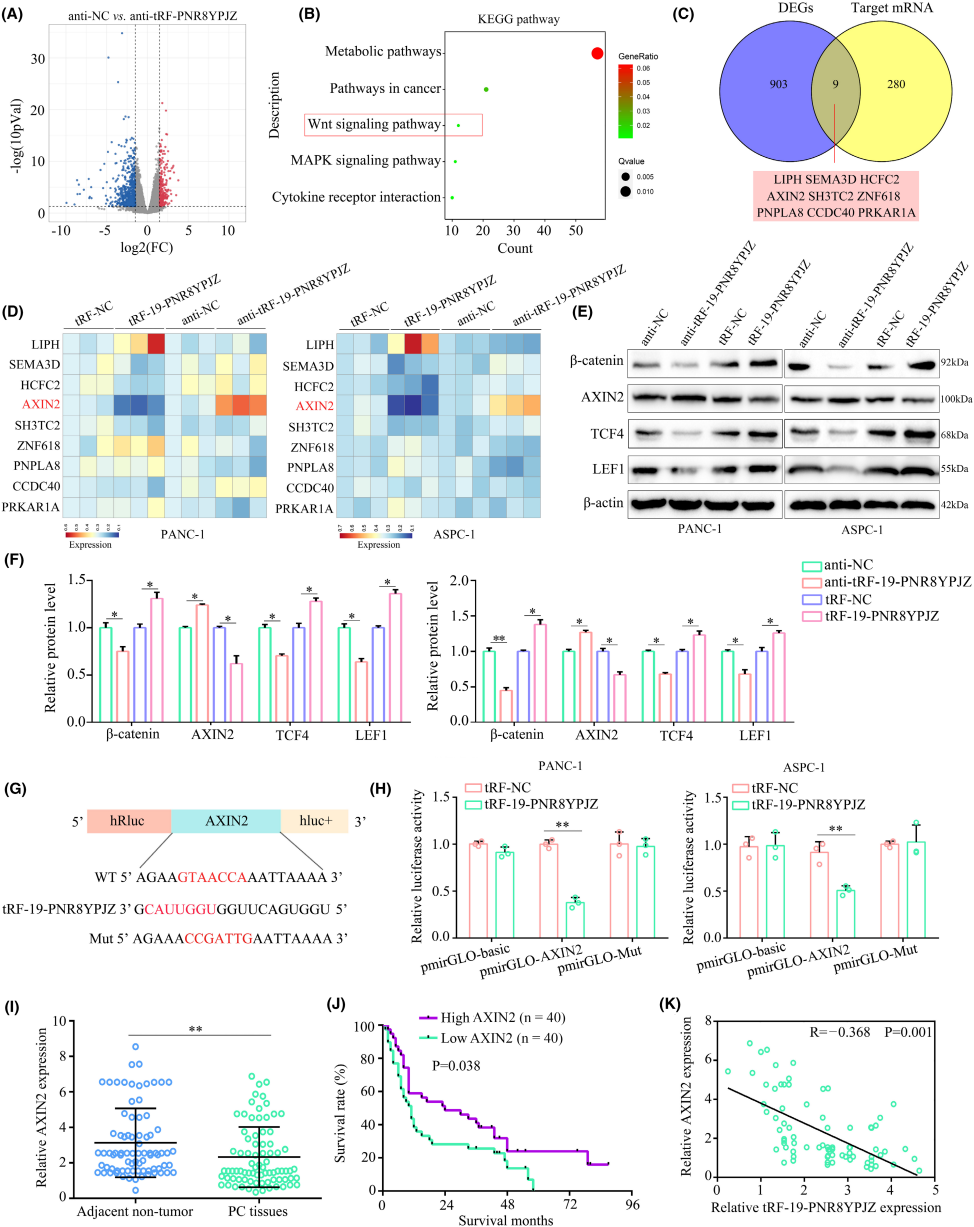
AXIN2 is a target of tRF‐19‐PNR8YPJZ in PC. (A) Pancreatic cancer cells (PANC‐1) were transfected with negative control (anti‐NC) and inhibitors of tRF‐19‐PNR8YPJZ (anti‐tRF‐19‐PNR8YPJZ) prior to the treatment with PSC‐derived exosomes. RNA sequencing was used to analyse the differently expressed proteins using a cut‐off as Log fold change >1.5 and *p* < 0.05. (B) KEGG analysis showed the top five enriched terms of differently expression proteins. (C) Venn diagram showing overlapping of the target mRNAs of tRF‐19‐PNR8YPJZ target prediction. (D) qRT‐PCR showed the mRNA levels of LIPH, SEMA3D, HCFC2, AXIN2, SH3TC2, ZNF618, PNPLA8, CCDC40, and PRKAR1A in PC cells transfected with anti‐tRF‐19‐PNR8YPJZ, tRF‐19‐PNR8YPJZ, or their negative controls. (E, F) Western blotting showed the protein levels of β‐catenin, AXIN2, TCF4, and LEF1 in PC cells transfected with anti‐ tRF‐19‐PNR8YPJZ, tRF‐19‐PNR8YPJZ, or their negative controls. (G) Schematic diagram of the predicted interaction position between tRF‐19‐PNR8YPJZ and the seed regions within the 3′UTR region and mutation region of AXIN2. (H) The luciferase activity of pmirGLO‐AXIN2 was significantly decreased by the tRF‐19‐PNR8YPJZ mimic in PC cells. (I) The expression level of AXIN2 was detected by qRT‐PCR in 80 paired PC and adjacent non‐cancerous tissues. (J) KM plot showed the overall survival in patients with low and high AXIN2 expression. (K) The Spearman rank correlation showed that negatively correlation with tRF‐19‐PNR8YPJZ and AXIN2. *p < 0.05; **p < 0.01.

The RNA of PC cells was sequenced to assess the molecular mechanisms involved in tRF‐19‐PNR8YPJZ. PANC‐1 cells knockdown tRF‐19‐PNR8YPJZ had 225 upregulated genes and 683 downregulated genes compared to those in control group (Figure 6A). According to KEGG analysis, these 912 differentially expressed genes (DEGs) were significantly enriched for metabolic pathways, cancer pathways, MAPK signalling pathways, Wnt signalling pathway and cytokine‐cytokine receptor interaction (Figure 6B, Table S2). Bioinformatic algorithms revealed eight potential target genes of tRF‐19‐PNR8YPJZ. Second, after overlapping the mRNA‐seq‐identified DEGs (*n* = 912) and the potential target genes of tRF‐19‐PNR8YPJZ predicted by bioinformatics databases (*n* = 289), nine intersection genes were identified, namely LIPH, SEMA3D, HCFC2, AXIN2, SH3TC2, ZNF618, PNPLA8, CCDC40 and PRKAR1A (Figure 6C). PC cells with tRF‐19‐PNR8YPJZ‐overexpression or inhibition showed the most significant reduction or increase in AXIN2 mRNA and protein levels (Figure 6D–F).

Next, we transfected PC cells with wild‐type and mutant AXIN2 fluorescent reporter plasmids based on their binding site genotypes (Figure 6G). We found that tRF‐19‐PNR8YPJZ reduced the fluorescence intensity in PC cells transfected with AXIN2 dual‐fluorescent reporter plasmids containing wild‐type binding sites, but not in those containing mutant binding sites (Figure 6H). In addition, the expression levels of AXIN2 in 80 paired PC tissues and adjacent tissues were determined using qRT‐PCR, and the mRNA levels of ANXIN2 were found to be elevated in PC tissues (Figure 6I). Furthermore, we found that low AXIN2 expression positively correlated with poor prognosis (Figure 6J). Moreover, we found that tRF‐19‐PNR8YPJZ expression was negatively associated with AXIN2 expression (Figure 6K). These results indicated that AXIN2 may be a target gene of tRF‐19‐PNR8YPJZ.

### 3.7 PSC‐derived exosomal tRF‐19‐PNR8YPJZ/AXIN2 axis promotes tumour progression

**FIGURE 7:**
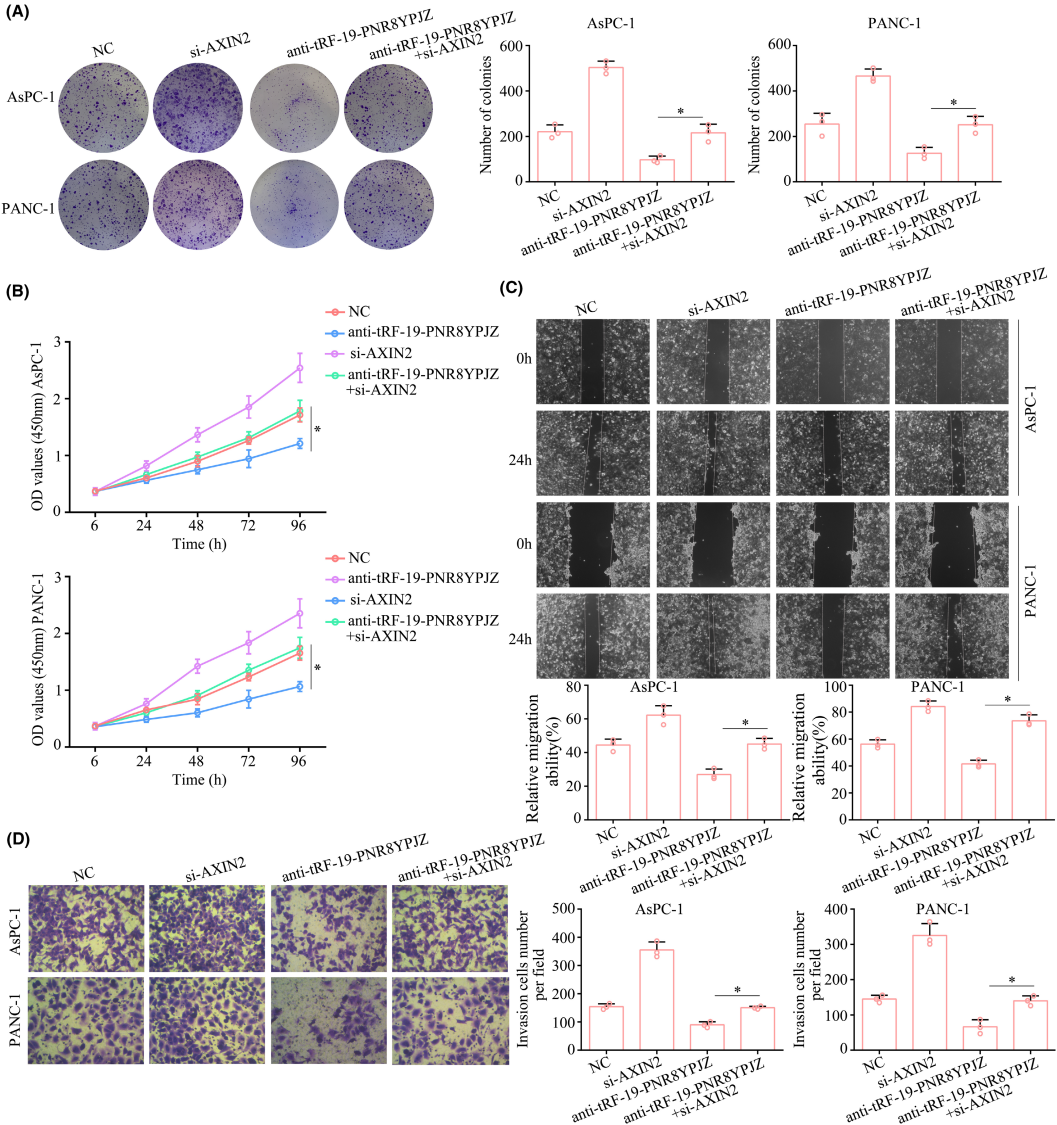
Restoration of AXIN2 reversed the inhibitory effects of tRF‐19‐PNR8YPJZ. Cells were divided into four groups and subjected to different treatments: negative control lentiviruses (NC); transfection of tRF‐19‐PNR8YPJZ knockdown lentiviruses (anti‐tRF‐19‐PNR8YPJZ) alone; treatment with AXIN2 small interfering RNA (si‐AXIN2) alone; transfection of anti‐tRF‐19‐PNR8YPJZ knockdown lentiviruses and treatment with AXIN2 small interfering RNA (si‐AXIN2+anti‐tRF‐19‐PNR8YPJZ) prior to treatment with PSC‐derived exosomes. (A) Colony formation assays were used to detect the cell proliferation. (B) CCK‐8 assays were used to detect the cell proliferation. (C) Wound healing assays were used to measure the migration of cells. (D) Transwell assays were used to measure the cell invasion. *p < 0.05.

Then, we investigated whether AXIN2 played a role in the biological functions of tRF‐19‐PNR8YPJZ in PC cells. According to CCK‐8 and colony formation assays, AXIN2 knockdown reversed the suppressive effects of anti‐tRF‐19‐PNR8YPJZ on cell viability and colony formation (Figure 7A,B). Additionally, anti‐tRF‐19‐PNR8YPJZ inhibited migration and invasion of PC cells in wound healing and transwell assays, but AXIN2 knockdown reversed this inhibition (Figure 7C,D).

## 4 DISCUSSION

Despite significant advancements in PC therapy, the prognosis of patients with PC remains poor.^16^, ^17^ Furthermore, most patients lost the optimal opportunity for therapy because of early metastasis.^18^ Therefore, identification of novel biomarkers may help the therapy of PC.

Evidence showed that PSC can secrete exosomes to promote the progression of PC cells. Herein, consistent with previous studies, we verified that the accelerative effects of PSC‐derived exosomes on PC cells proliferation, migration and invasion. Non‐coding RNAs were key parts of content in PSC‐derived exosomes. Previous studies indicated that non‐coding RNA^10^, ^16^ in PSC‐derived exosomes is a key mediator to promote the progression of PC. For example, Chi et al. exhibited that exosomal lncRNA UCA1 derived from PSCs increased the resistance of gemcitabine of PC.^19^ PSC‐derived exosomes Li et al. suggested that PSC‐derived exosomes can submit miR‐5703 to PC cells and increased PC cell proliferation and metastasis.^10^ Unlike previous studies concentration on LncRNAs and miRNAs, we focused on a novel class of non‐coding RNAs, named tRNA‐derived small RNAs. Through sequencing, tRF‐19‐PNR8YPJZ had aroused our great concern, while its expression was significantly increased in the PC cells after treatment with PSC‐derived exosomes.

tRNA‐derived small RNAs (tDRs) are fragments of precursor or mature tRNAs that are usually 14–50 nucleotides (nt) in length. Previous studies indicated that tDRs can serve as diagnostic and prognostic biomarkers for cancers. For example, while 5′‐tRF‐GlyGCC expression was elevated in colon cancer tissues and blood samples, it can serve as prognostic biomarker.^20^ Similarly, tDR‐0009 and tDR‐7336 was increased in cells under hypoxia, and predicted doxorubicin resistance in triple‐negative breast cancer.^21^ However, key tDRs for PC were still unknown. After reviewing of clinical characteristics, we found that the expression of exosomal tRF‐19‐PNR8YPJZ was also increased in patients with PC compared to that in matched non‐cancerous tissues. High expression of tRF‐19‐PNR8YPJZ was also associated with advanced clinical stage, perineural invasion, lymph node invasion and poor prognosis. Therefore, we considered that the tRF‐19‐PNR8YPJZ is a distinguish biomarker for PC diagnosis and prognosis.

tDRs have been identified as vital regulators of biological function and have been shown to play oncogenic or suppressor roles in different tumours by decreasing the expression of target genes.^22^ Through their interaction with proteins or mRNA, inhibition of translation and regulation of gene expression, tRFs play biological roles.^23^ It has also been observed that dysregulation of tDRs is associated with several cancers, including PC.^24^, ^25^, ^26^ tRFs are part of tDRs and formed by the processing of tRNA by nucleases such as DICER and angiopoietin under certain conditions.^27^, ^28^ In addition to the simple transport function, tRFs can regulate gene expression through transcriptional, post‐transcriptional and epigenetic approach.^29^, ^30^ It has been found that the 5′ tiRNA‐His‐GTG levels are elevated in colorectal cancer, whereas the levels of 5′ tiRNA‐His‐GTG are correlated with the size of the tumour.^31^ In addition, tsRNA‐MetCAT‐37 and tsRNA‐ValTAC‐41 levels were elevated in PC tissues, which were used to distinguish PC tissues from adjacent non‐tumour tissues.^32^ In the current study, we found that PSC‐derived exosomes increased the proliferation and metastasis of PC cells, while knockdown of tRF‐19‐PNR8YPJZ in PC cells relieved the stimulative effects of PSC‐derived exosomes. Therefore, our study identified that exosomal tRF‐19‐PNR8YPJZ was an oncogenic mediator in PSC‐derived exosomes to promote PC cell proliferation and metastasis.

AXIN2 is a homologous protein of the Axin family and has a high degree of structural and functional similarity with AXIN.^33^ AXIN2, a negative inhibitor of the Wnt signalling pathway, regulates the phosphorylation and degradation of β‐catenin, thus promoting the overexpression of target genes, affecting cell proliferation and differentiation, and promoting the occurrence of tumours.^34^, ^35^, ^36^ In this study, we showed that tRF‐19‐PNR8YPJZ bound to AXIN2 and that overexpression of this receptor activated the Wnt/β‐catenin pathway by decreasing AXIN2 expression. Knockdown of tRF‐19‐PNR8YPJZ prior to PSC‐derived exosome treatment reversed its effects on Wnt/β‐catenin pathway activation.

Taken together, the present study demonstrated that PSC‐derived exosomal tRF‐19‐PNR8YPJZ was delivered to PC cells and promoted proliferation and mobility via AXIN2. Exosomal tRF‐19‐PNR8YPJZ may be a potential biomarker for the diagnosis of PC and an effective target for clinical therapy. Therefore, it is expected to develop nucleic acid drugs targeting tRF‐19‐PNR8YPJZ in the future to treat PC. However, the stability of nucleic acid drugs is generally poor, and the development of related targeted delivery systems is not perfect. There is still a long way to go for tRF‐19‐PNR8YPJZ to be used in clinical treatment.

## 5 CONCLUSION

PSCs‐derived exosomal tRF‐19‐PNR8YPJZ promotes the malignant activity of PC by regulating AXIN2.

## AUTHOR CONTRIBUTIONS

**Wenpeng Cao:** Data curation (equal); funding acquisition (equal). **Shisi Dai:** Data curation (equal); resources (equal). **Wanyuan Ruan:** Data curation (equal). **Tingting Long:** Formal analysis (supporting). **Zhirui Zeng:** Methodology (equal). **Shan Lei:** Conceptualization (equal); writing – original draft (equal).

## FUNDING INFORMATION

This study was supported by the National Natural Science Foundation of China (82160567), Education Department of Guizhou Province Project [No. YJSCKYJJJH (2021) 143].

## CONFLICT OF INTEREST STATEMENT

The authors declare that they have no competing interests.

## CONSENT FOR PUBLICATION

All authors have agreed to publish this manuscript.

## Supporting information


Figure S1.

Figure S2.

Table S1.

Table S2.

## Data Availability

The datasets used and/or analysed during the current study are available from the corresponding author on reasonable request.
