# Supplementary figures and images for: Pancreatic stellate cell‐derived exosomal tRF‐19‐PNR8YPJZ promotes proliferation and mobility of pancreatic cancer through AXIN2
Source: J Cell Mol Med. 2023 Jul 24;27(17):2533–46. doi: 10.1111/jcmm.17852 (PMC10468654; doi:10.1111/jcmm.17852)

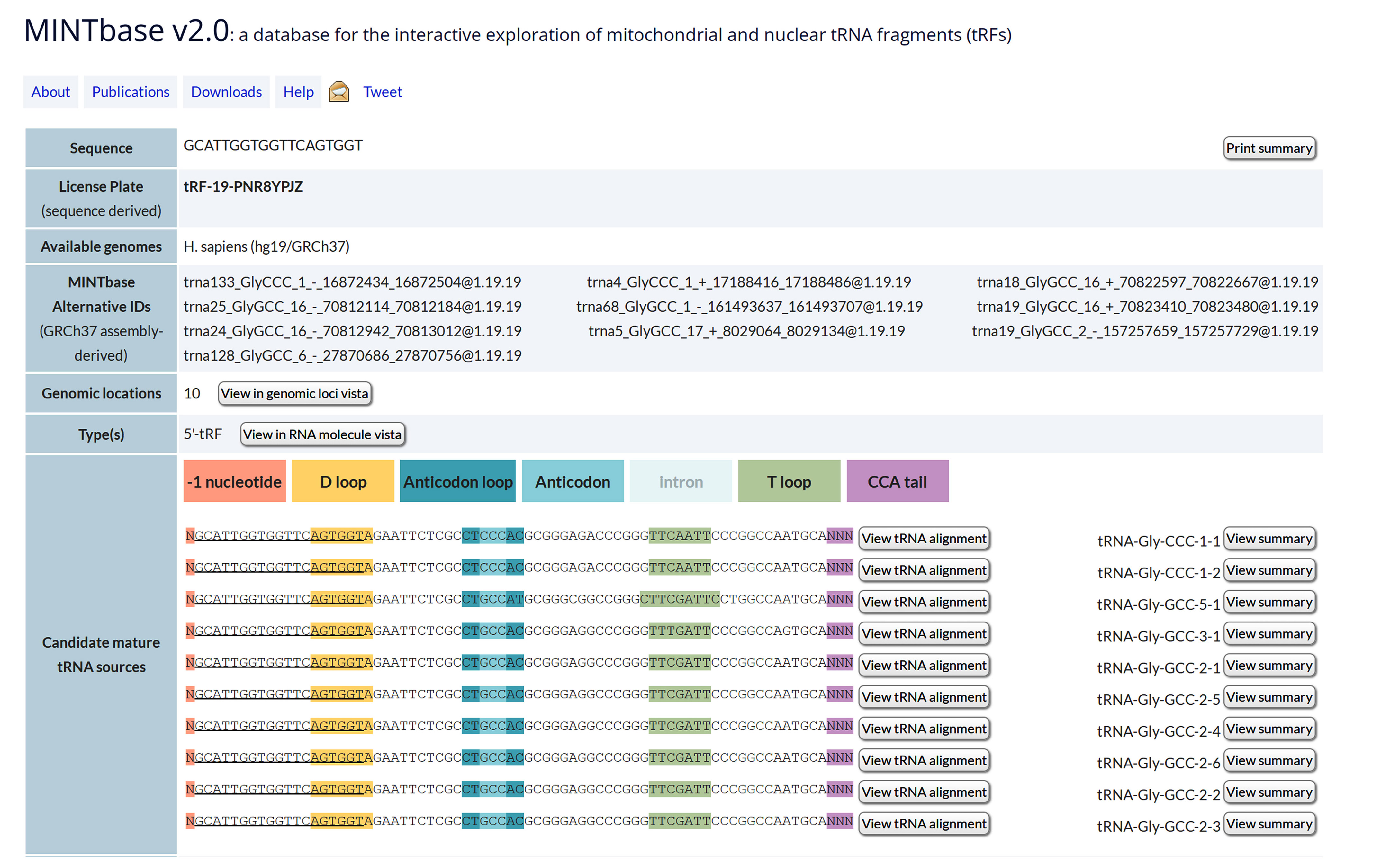

Supplement: Supplementary file 1 — Figure S1. [file JCMM-27-2533-s002.tif]

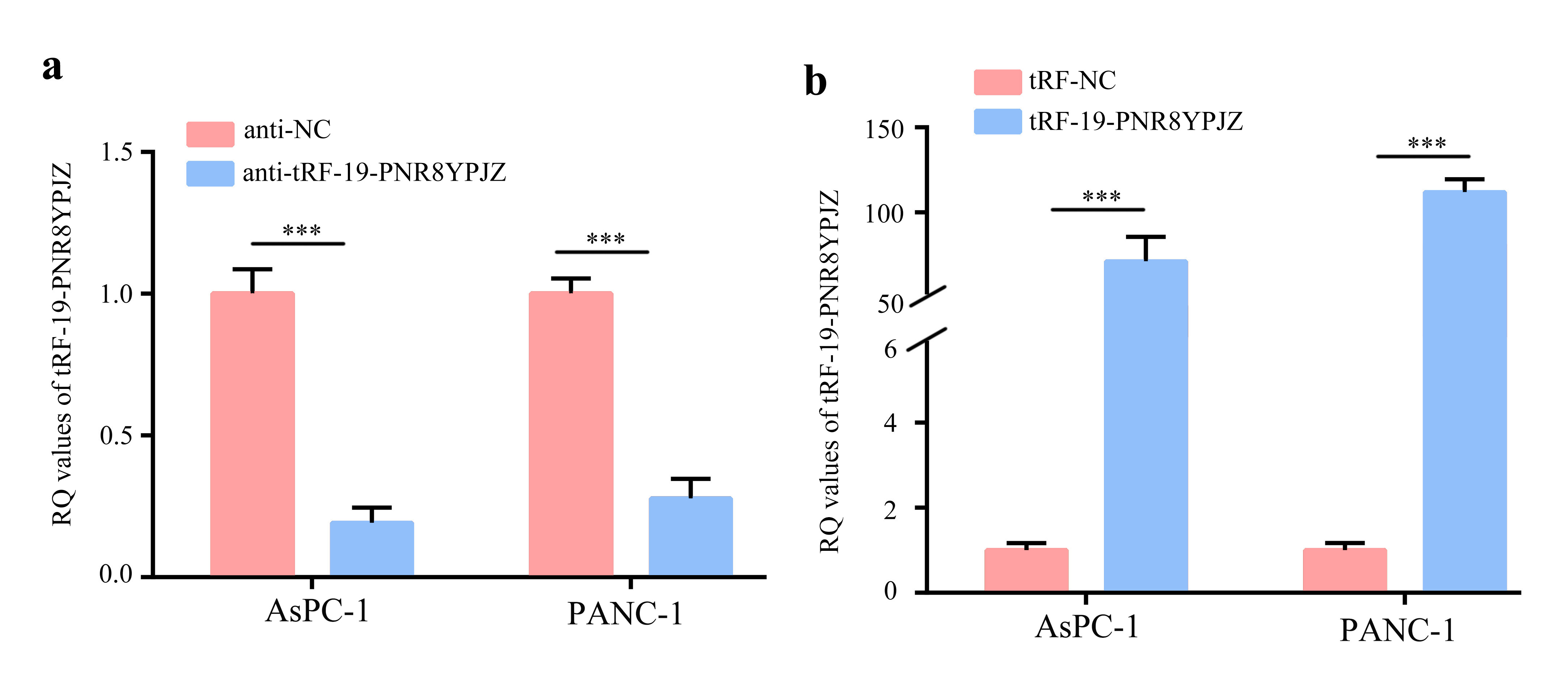

Supplement: Supplementary file 2 — Figure S2. [file JCMM-27-2533-s003.tif]
